# Coloured ornamental traits could be effective and non-invasive indicators of pollution exposure for wildlife

**DOI:** 10.1093/conphys/cow028

**Published:** 2016-08-26

**Authors:** Natalia Lifshitz, Colleen Cassady St Clair

**Affiliations:** Department of Biological Sciences, University of Alberta, Z-708, 11455 Saskatchewan Drive, Edmonton, Alberta, CanadaT6G 2E9

**Keywords:** Behavioural ecology, chemical pollution, coloured ornamental traits, conservation biology, non-invasive monitoring, review

## Abstract

Growth in human populations causes habitat degradation for other species, which is usually gauged by physical changes to landscapes. Corresponding habitat degradation to air and water is also common, but its effects on individuals can be difficult to detect until they result in the decline or disappearance of populations. More proactive measures of pollution usually combine abiotic samples of soil, water or air with invasive sampling of expendable species, but this approach sometimes creates ethical dilemmas and has limited application for threatened species. Here, we describe the potential to measure the effects of pollution on many species of birds and fish by using ornamental traits that are expressed as coloured skin, feathers and scales. As products of sexual selection, these traits are sensitive to environmental conditions, thereby providing honest information about the condition of their bearers as ready-made biomarkers. We review the documented effects of several classes of pollutants, including pharmaceuticals, pesticides, industry-related compounds and metals, on two classes of colour pigments, namely melanins and carotenoids. We find that several pollutants impede the expression of both carotenoids and brown melanin, while enhancing traits coloured by black melanin. We also review some of the current limitations of using ornamental colour as an indicator of pollution exposure, suggest avenues for future research and speculate about how advances in robotics and remote imagery will soon make it possible to measure these traits remotely and in a non-invasive manner. Wider awareness of this potential by conservation managers could foster the development of suitable model species and comparative metrics and lay a foundation for pollution monitoring that is more generalizable and biologically relevant than existing standards.

## Abstract (precis)

Sub-lethal impacts of environmental pollution are difficult to detect and usually require invasive methods. We review the evidence that changes in ornamental colour in fish and birds can be associated with a variety of pollutants. Although these ornaments evolved for mate choice, they could also provide a non-invasive indicator of pollution exposure.

## Introduction

In the field of conservation biology, anthropogenic changes to the environment are widely known to contribute to population declines and extinctions of vulnerable species (reviewed by Gibbons *et al*., 2000). In the field of behavioural ecology, ornamental traits are widely understood to be reliable indicators of individual condition and quality (Zahavi, 1975; Hamilton and Zuk, 1982) that consistently reflect environmental conditions (Griffith *et al*., 1999; Monaghan, 2008). Despite extensive independent development and some appreciation of their combined explanatory potential (Hill, 1995; Sutherland, 1998), these two bodies of theory are almost never combined by conservation biologists and wildlife managers. As suggested by Hill (1995), better integration of these fields could provide a general, proactive, non-invasive and effective diagnostic tool for detecting subtler anthropogenic effects on individuals before they are signalled by decreased reproduction or higher probability of mortality, which ultimate cause declining populations. In turn, those advantages could reduce the cost and increase the efficacy of mitigation efforts (Clout *et al*., 2002; Buchholz, 2007).

Estimation of anthropogenic effects using ornamental traits requires mechanistic understanding of both anthropogenic effects and the expression of ornamental traits in wildlife. Separately, these mechanisms are particularly developed for one category of each component; chemical pollution as a type of anthropogenic effect, and the pigment-based colouration of the integument as a type of ornamental trait. In this review, we integrate these two specific categories to illustrate how they could be used by conservation biologists now, describe some limitations for expanding that use, and identify profitable directions for future research.

Chemical pollution can have acute negative effects on wildlife via development (Colborn *et al*., 1993), physiology (Ross *et al*., 1994), behaviour (Zala and Penn, 2004), reproductive success (Goutte *et al*., 2014) and survival (Martínez-Abraín *et al*., 2006). It can also increase rates of hybridization (Ward and Blum, 2012) and decrease genetic diversity (Bickham *et al*., 2000), ultimately reducing population viability and leading to extinction (Kidd *et al*., 2007). All of these effects may be foretold by changes to the conspicuously coloured ornamental traits that are prevalent in species with strong mate selection, such as some birds and fish. As proposed by Darwin (1871), ornamental traits are morphological or behavioural traits that evolved via sexual selection to confer reproductive, rather than survival, advantages to the bearer through enhanced ability to attract mates. An example of this is the flamboyant tail of the male peacock. Zahavi (1975) expanded Darwin's theory to propose that such traits are so costly to their bearers that they can be produced and maintained as handicaps only by individuals of greater quality and condition. As such, ornamental traits are now widely recognized to reveal good genes, developmental conditions, local environments at the time when such traits are developed, or all three (von Schantz *et al*., 1999).

Ornamental traits reveal the quality and condition of their bearers partly because they have high phenotypic plasticity (Cotton *et al*., 2004), meaning that their expression is particularly sensitive to the environment and to the cascade of physiological mechanisms produced by stressful events (Hill, 1995; Buchanan, 2000). Environmental conditions can be canalized during development (e.g. Naguib and Nemitz, 2007), but they can also be reflected in a more recent or current fashion (e.g. Velando *et al*., 2006). This plasticity and honesty make ornamental traits especially useful for detecting both temporal and spatial variation in environmental conditions.

A prevalent type of ornamental trait in vertebrates is integument colouration (Hill and McGraw, 2006b), which includes skin (e.g. Velando *et al*., 2006), scales (e.g. Plasman *et al*., 2015), hair (e.g. West and Packer, 2002) and feathers (e.g. Safran and McGraw, 2004). The appearance of the integument is dependent on its structure and the pigments deposited inside it as well as by the dirt, waxes and abrasion applied to or acquired by it over time (Hill and McGraw, 2006a). At least four kinds of costs can be revealed by these traits. First, the pigments involved in trait colouration may be physiologically costly to produce, such as melanin (Jawor and Breitwisch, 2003). Second, the pigments may be available only in some kinds of high-quality foods, such as carotenoids (McGraw, 2006a). Third, the pigments may be needed for other functions, such as immune or antioxidant system support, and available for ornaments only when those functions have been met (Faivre *et al*., 2003; Galván and Alonso-Alvarez, 2008). Finally, each of these costs may co-occur as additive or multiplicative effects. By any of these routes, only those individuals in good condition can afford to produce, acquire or allocate pigments for trait colouration without compromising survival, making colouration an honest signal to observers (Hamilton and Zuk, 1982; Grafen, 1990).

The ubiquity, plasticity and honesty of integument colouration make it a powerful indicator of the competing costs of environmental stressors, such as anthropogenic pollutants. The short temporal scale of environmental effects on the integument suggests the potential of using integument colouration as a tool to detect negative environmental impacts on individuals long before they cascade through populations, communities and ecosystems with effects that are more difficult and costly to reverse. These features give ornamental integument colouration enormous but largely untapped potential to diagnose many conservation problems at their most proximate stages, in order to support solutions that are more proactive (e.g. Baruch-Mordo *et al*., 2013), generalizable (*sensu* Caughley, 1994) and holistic (Caro and Sherman, 2013).

**Figure 1:**
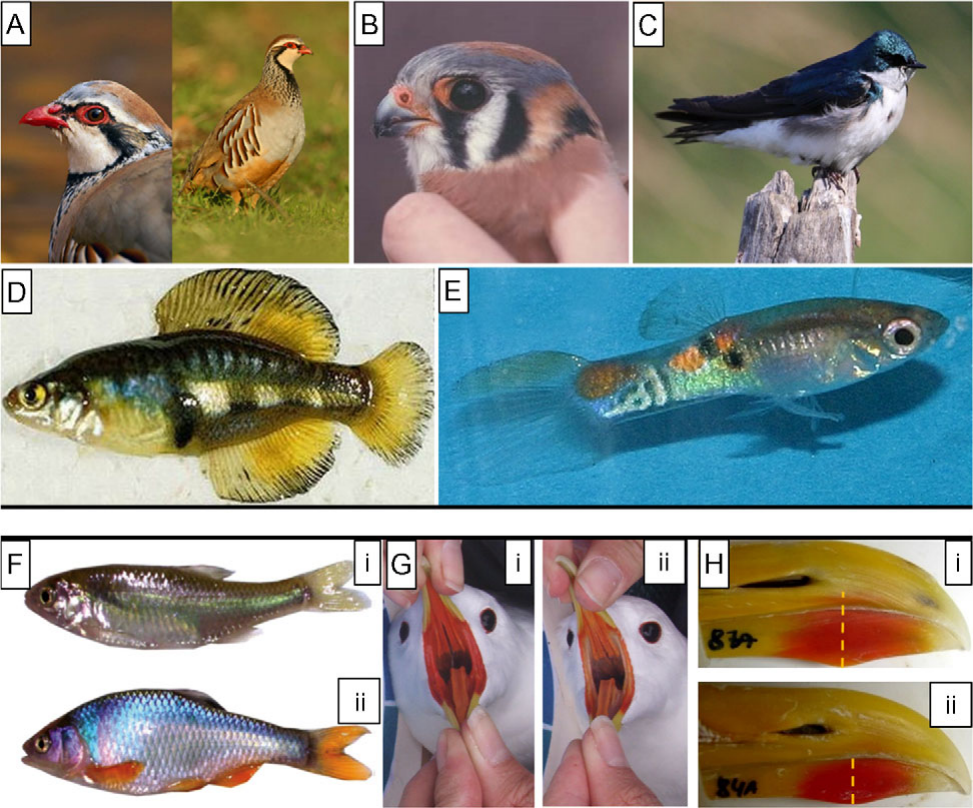
Upper panel provides examples of coloured ornamental traits that show evidence of pollution. (**A**) Red colouration of the facial skin, brown and black flank feather bands and black feather bib of red-legged partridges (Galván and Alonso-Alvarez, 2009; Alonso-Alvarez and Galván, 2011) Photographs from Alonso-Alvarez and Galván (2011). (**B**) Yellow colouration of the facial skin of American kestrels (Bortolotti *et al*., 2003). Photographs by Gary Bortolotti and Russell D. Dawson, courtesy of Kim Fernie. (**C**) Iridescent blue–green plumage of tree swallows (McCarty and Secord, 2000). Photograph by Natalia Lifshitz. (**D**) Yellow colouration of fins and tale of amarillo fish (Arellano-Aguilar and Macías Garcia, 2008). Photograph by Shane Webb, courtesy of Constantino Macías García. (**E**) Body yellow–orange spots of guppies (Toft and Baatrup, 2001; Kristensen *et al*., 2005; Shenoy, 2012). Photograph by Erik Baatrup. Lower panel provides examples of changes in ornamental colouration of fish and birds exposed to anthropogenic pollutants. (**F**) Ornamental body colouration of adult male red shiner (*Cyprinella lutrensis*) exposed to the oestrogen 17β-estradiol (**i**) and control water (**ii**). (McGree *et al*., 2010). (**G**) Gape and tongue colouration of female black-legged kittiwakes (*Rissa tridactyla*) with low (**i**) or high concentrations of pesticides and PCBs in blood (**ii**; Blévin *et al*., 2014). Photographs by Olivier Chastel. (**H**) Red bill spot (controlled by bill size) of yellow legged male gulls (*Larus michahellis*) experimentally fed with oil from the Prestige oil spill (**i**) or control sunflower oil (**ii**; Pérez *et al*., 2010). Photographs by Cristobal Pérez.

In the following sections, we describe the two main types of pigment-based colouration found in ornamental integuments of vertebrates, melanins and carotenoids, and review the known effects on them of a variety of chemical pollutants (Fig. 1 and Table 1). Existing research in this area has focused only on fish and birds, groups that frequently express quantifiable coloured integuments and so have well-developed literatures. The Latin names for the species we refer to in the text are provided in Table 1. Following this review, we discuss the limitations to the use of colouration as an indicator of pollution exposure and propose some recommendations for future research.

**Table 1:** Summary of the effects of chemical pollutants on coloured ornamental traits of fish and birds

Product, use and concentration	Suggested pathway	Pigment	Affected trait and direction of effect	Age class, sex and Latin name	Reference
Pharmaceuticals and active ingredients in personal care products					
E2/natural oestrogen/AN	ED	Car	Area and colour of body orange spots (−)	Adult male guppies (*Poecilia reticulata*)	Toft and Baatrup (2001)
EE2/artificial oestrogen/AN	ED	Car	Area of body orange spots (−)	Adult male guppies (*P. reticulata*)	Kristensen *et al*. (2005)
EE2/artificial oestrogen/WN	ED	Car	Reddish body colouration (−)	Adult male zebrafish (*Danio rerio*)	Larsen *et al*. (2008)
E2/natural oestrogen/WN	ED	Car	Colouration of pectoral and caudal fins (−)	Adult male red shiners (*Cyprinella lutrensis*)	McGree *et al*. (2010)
Pesticides					
Vinclozolin/fungicide and *p*,*p*′-DDE/principal metabolite of the insecticide DDT	ED	Car	Area and colour of body orange spots (−)	Adult male guppies (*P. reticulata*)	Baatrup and Junge (2001)
Methyl parathion/insecticide/WN	Damage to embryo's physiology	Car	Colour of yellow fins and body (−)	Adult male amarillo fish (*Girardinichthys multiradiatus*)	Arellano-Aguilar and Macías Garcia (2008)
Atrazine/herbicide/WN	ED	Car	Area of orange spots (−)	Adult male guppies (*P. reticulata*)	Shenoy (2012)
Diquat/contact herbicide	OS	Mel	Area of black (+) and brown (−) plumage patches	Adult male red-legged partridges (*Alectoris rufa*)	Galván and Alonso-Alvarez (2009)
Diquat/contact herbicide	OS	Car	Colour of red beak and eye rings (−)	Adult male red-legged partridges (*A. rufa*)	Alonso-Alvarez and Galván (2011)
Thiram and difenoconazole/fungicides and imidacloprid/insecticide/WN	OS	Car	Area of red eye ring (−)	Adult male red-legged partridges (*A. rufa*)	Lopez-Antia *et al*. (2013)
Mix of pesticides and PCBs	OS (potentially)	Car	Colour of orange–red eye ring, gapes and tongue (−)	Adult female black-legged kittiwakes (*Rissa tridactyla*)	Blévin *et al*. (2014)
Industry-related compounds					
Octylphenol/AN	ED	Car	Colour and size of orange spots (−)	Adult male guppies (*P. reticulata*)	Toft and Baatrup (2001)
Bisphenol A	ED	Car	Body colour intensity (−)	Adult male red shiners (*Cyprinella lutrensis*)	Ward and Blum (2012)
PCBs (mix)	ED	Mel	Onset of plumage maturation (+)	Subadult female tree swallows (*Tachycineta bicolour*)	McCarty and Secord (2000)
Aroclor (PCB)/WN	ED	Car	Colour of yellow facial skin (−)	Adult male American kestrels (*Falco sparverius*)	Bortolotti *et al*. (2003)
PAHs (mix)/WN	OS	Car	Size of red bill spot (−)	Adult male and female yellow-legged gulls (*Larus michahellis*)	Pérez *et al*. (2010)
Metals					
Lead, cadmium, zinc and copper	OS/carotenoid sources	Mel	Area of black breast stripes (+) and colour of yellow breast feathers (−)	Young and adult male and female great tits (*Parus major*)	Dauwe and Eens (2008)
Mercury	OS, health and/or pigment production	Mel	Brightness of blue chest feathers (+)	Adult male and female belted kingfishers (*Megaceryle alcyon*)	White and Cristol (2014)
Sulphuric oxides, copper, zinc, nickel and lead	OS/carotenoid sources	Car	Intensity of yellow breast feathers (−)	Nestlings of great tit (*P. major*)	Eeva *et al*. (1998)
Cadmium, lead, arsenic, copper and zinc	OS/carotenoid sources	Car	Colour of yellow breast feathers (−)	Young and adult male and female great tits (*P. major*)	Geens *et al*. (2009)

For experimental studies, the concentration is indicated, when available, as within natural levels (WN) or above natural levels (AN). The pollutants included act via two main suggested physiological effects, oxidative stress (OS) and endocrine disruption (ED); other possible causes are indicated. Effects of pollutants on ornamental traits are further categorized by type of pigment [melanins (Mel) and carotenoids (Car)], and their expression in coloured, integumentary traits of fish and birds. Each example also names the product and use, the affected trait, age class, sex and scientific name of the studied species, and the associated reference. Abbreviations: DDT, dichlorodiphenyltrichloroethane; PAHs, polycyclic aromatic hydrocarbons; PCBs, polychlorinated biphenyls; E2, 17β-estradiol; EE2, 17α-ethinylestradiol; p,p′-DDE, dichlorodiphenyldichloroethylene.

## Overview of pigment and pollutant types

Melanins are the most prevalent pigments in vertebrates, producing many yellow–brownish (pheomelanin) and grey–black (eumelanin) traits (reviewed by McGraw, 2005). They can also combine with keratin and air to produce structural colours, such as blue, violet, green and ultraviolet hues, as well as iridescent colours (Prum, 2006). As vertebrates can synthesize melanins *de novo* from amino acid precursors (Lin and Fisher, 2007), they are frequently assumed to be an unlimited resource for ornamental trait building, strictly controlled by genes (e.g. Badyaev and Hill, 2000; McGraw, 2006b). However, more recent studies have demonstrated that the expression of melanin-based ornaments can also be influenced by environmental factors, such as rearing conditions, parasite infestation and diet quality, to give these ornaments a plastic and honest quality in vertebrates (Fargallo *et al*., 2007; reviewed by McGraw, 2008; Guindre-Parker and Love, 2014).

Despite the ubiquity of melanin in vertebrates, a second kind of pigment, namely the carotenoids, are more widely studied. They produce many yellow, orange and red traits, but cannot be synthesized by vertebrates (Schiedt, 1989), so their acquisition through the diet makes them a limited resource. Moreover, carotenoids are thought to play key physiological functions, accepting free radicals to protect cells and tissues from oxidative damage and acting as immune system enhancers (reviewed by Lozano, 1994; von Schantz *et al*., 1999; but see Hartley and Kennedy, 2004; Pérez-Rodríguez, 2009). In combination, these properties provide information about animal foraging, carotenoid uptake and allocation efficiency that is both accurate and visible (McGraw, 2006a).

Both types of pigments are pertinent to the large literature addressing the negative physiological effects of pollution on wildlife either by disrupting the endocrine system (Tyler *et al*., 1998) and/or by altering the oxidative status of individuals (Isaksson, 2010). Endocrine-disrupting compounds interfere with the normal function of the endocrine system in several ways, such as mimicking natural hormones, blocking or altering the binding of natural hormones to hormone receptors, altering production and breakdown of natural hormones and altering the production and function of hormone receptors (Tyler *et al*., 1998). These effects have the potential to impact ornamental colouration if its expression is controlled by or related to sexual hormones (e.g. Bókony *et al*., 2008).

The effect of pollution on the oxidative status of individuals is subtler, but it may also be more ubiquitously and generally expressed. Metabolism produces reactive oxygen species as a byproduct, and when this production exceeds the capacity of the antioxidant defense and repair mechanisms, an imbalance called oxidative stress arises, which leads to oxidative damage to biomolecules (i.e. proteins, lipids and DNA; Halliwell and Gutteridge, 2007). This imbalance is known to be a key component in the life-history trade-offs between growth, reproduction and self-maintenance or survival (Monaghan *et al*., 2009). Therefore, animals exposed to pollutants with pro-oxidant properties and whose ornamental colouration depends upon pigments related to antioxidant defenses are faced with a trade-off between using antioxidants to combat oxidative stress or to signalling functions and, ultimately, may have to sacrifice their investment in signalling to support survival.

The categorization of pollutants is confusing because it sometimes refers to generalized effects on natural systems (e.g. endocrine-disrupting compounds), sometimes to the chemical nature of compounds [e.g. polycyclic aromatic hydrocarbons (PAHs)] and sometimes to the originating industries (e.g. petroleum products). In this review, we simplify that categorization by referring consistently to the anthropogenic context of pollutants and review examples of effects on ornament colouration for each of the following: pharmaceuticals and active ingredients in personal care products (PPCPs); pesticides; industrial pollutants that are prevalent but not restricted to petroleum production; and heavy metals that frequently result from mining and manufacturing. Within each of these source categories, we review the known effects on the production and expression of melanins and on the uptake and use of carotenoids for ornament colouration (Fig. 1 and Table 1).

## Pharmaceuticals and active ingredients in personal care products

This category conventionally includes prescription and non-prescription drugs, oral contraceptives, fragrances and cosmetics (Daughton and Ternes, 1999). These ubiquitous products are usually disposed of or discharged into the environment on a continual basis via domestic and industrial sewage systems and wet-weather runoff, where they enter water courses and come into direct contact with aquatic organisms. Relative to other pollutants, the emission of pharmaceutical products tends to be chronic and concentrated in areas with high human density (Kolpin *et al*., 2002). Given that some PPCPs (such as oral contraceptives) are specifically designed to target and modulate the endocrine system, they are likely to affect any form of ornamental colouration that is controlled by it.

Despite the high potential for PPCPs to influence ornamental colouration, this has been addressed only in fish, and no study has targeted birds. To date, there is no evidence of negative effects of PPCPs on melanin-based colouration, where likely effects include ornamental traits that are mediated and stimulated by hormones, such as androgens or thyroid hormones (reviewed by McGraw, 2006b).

The anticipated effects of exogenous oestrogens on aquatic species have been robustly demonstrated with fish. In a laboratory study, exposure to the artificial oestrogen 17α-ethinylestradiol (EE2) reduced the area of carotenoid-orange colouration as a percentage of the total body area of male guppies (Kristensen *et al*., 2005), a characteristic known to function as a sexually selected signal to females (Kodric-Brown and Nicoletto, 2001). In addition, exposed male guppies showed a significant reduction in courtship behaviour, sperm count and paternity when competing for fertilizations with unexposed males (Kristensen *et al*., 2005). Similar effects occurred in male guppies, zebra fish and red shiners that were exposed in the laboratory to the natural oestrogens 17β-estradiol (E2), EE2 and E2, respectively, and then expressed dampened mating colouration (Toft and Baatrup, 2001; Larsen *et al*., 2008; McGree *et al*., 2010). Treated male guppies produced fewer offspring and displayed paler ornaments even after 3 months of recovery in clean water (Toft and Baatrup, 2001). Male zebra fish also showed a significant reduction in courtship behaviours, failing to induce spawning in females (Larsen *et al*., 2008). Likewise, courtship behaviours of male shiners were significantly reduced, as was their fertilization success, causing null hatching success of the fertilized eggs. Importantly, when exposure ceased, other reproductive end points, but not colouration, of the shiners improved significantly, demonstrating that the dulling effects were longer lasting (McGree *et al*., 2010).

## Pesticides

Pesticides are a broad category of compounds that are commonly used to protect crops, livestock, domestic animals and humans from damage and diseases caused by fungi (fungicides), insects (insecticides), rodents (rodenticides), competition with unwanted plants (herbicides) and other so-called pests. Pesticides can also be categorized according to their chemical structure into inorganic (compounds that contain arsenic, copper, lead or mercury) and organic chemicals [artificially synthesized chemicals, such as dichlorodiphenyltrichloroethane (DDT); reviewed by Freedman, 2001]. Animals may be exposed to pesticides through consumption of contaminated food or water, as well as through inhalation of contaminated air. Although pesticides have been used for millennia, their use has increased greatly during the past half-century because of their economic benefits and increased worldwide availability (Pimentel *et al*., 1992). This has increased the geographical and temporal risk of exposure for wide-ranging and migratory birds (Stutchbury, 2009) among other species. Pesticides primarily cause damage to organisms by producing free radicals that overwhelm the antioxidant system (Abdollahi *et al*., 2004), but some compounds also alter the endocrine system (Colborn *et al*., 1993).

Effects on melanin-based colouration can be complex and counterintuitive, as has been shown in red-legged partridges exposed to the contact herbicide diquat. In these birds, the eumelanic (black) plumage of adults was unexpectedly enlarged following exposure to the pesticide during development, to produce larger black-spotted bibs and black flank bands (Galván and Alonso-Alvarez, 2009), which signal higher-quality individuals of both sexes (Bortolotti *et al*., 2006). However, exposure to the herbicide simultaneously reduced expression of pheomelanin to cause the brown flank bands to be smaller (Galván and Alonso-Alvarez, 2009). The authors speculated that the pesticide increased oxidation, which depleted the intracellular antioxidant glutathione and reduced the amount available to produce brown pigment in the flank bands. Given that circulating glutathione blocks eumelanin synthesis, its depletion also caused the larger black bibs and flank bands (Galván and Alonso-Alvarez, 2009). This study underscores the need to understand underlying physiological processes to interpret the effects on colouration of exposure to pollution.

The negative effects of pesticides on carotenoid-based ornamental colouration have been robustly demonstrated in fish. For example, the consistent preference by female guppies for males with larger and more intense orange spots (described above) clearly predicts a disadvantage to males exposed to the fungicide vinclozolin and to the principal metabolite of the insecticide DDT [*p*,*p*′-dichlorodiphenyldichloroethylene (DDE)] because they reduced both the size and intensity of the spots (Baatrup and Junge, 2001). These morphological changes also occurred when guppies were exposed to the herbicide atrazine, with corresponding reductions in courtship displays and aggressive behaviour toward other males during competition for mates (Shenoy, 2012).

The effects of pollutants on adult colouration can also result from exposure during development. The sexually selected yellow colouration of adult male Amarillo fish (Macías Garcia, 1991) was duller in males exposed as embryos to the insecticide methyl parathion and resulted in lower rates of female visitation and copulation attempts during courtship (Arellano-Aguilar and Macías Garcia, 2008). The authors speculated that these effects in adults could have been caused by one or both of long-lasting damage to the physiological systems that process and deposit carotenoids, or permanent damage to their antioxidant system with resulting increases in the use of dietary carotenoids to combat oxidative stress during adulthood.

In some cases, the interacting and opposing effects of pollutants on different colourful ornaments may help to identify basic physiological pathways and reveal the detrimental mechanisms of pollution exposure. An example of this potential is provided by the red-legged partridges (described above), in which the diquat-exposed birds with enhanced eumelanic traits also had paler carotenoid-based ornaments in the form of red beak and eye rings (Alonso-Alvarez and Galván, 2011). Given that the red colouration in the head integument is positively correlated with the health status of individuals (Mougeot *et al*., 2009) and positively affects female reproductive investment (Pérez-Rodríguez and Viñuela, 2008), researchers could see that the net effects of pesticide exposure were negative, despite the increase in eumelanin expression (Alonso-Alvarez and Galván, 2011). These negative effects on the carotenoid-based colouration of red-legged partridges were later confirmed for two fungicides (thiram and difenoconazole) and an insecticide (imidacloprid) via reductions in the percentage of carotenoid pigmentation in the eye ring. As additional evidence of net negative effects, all three pesticides reduced the size of eggs, imidacloprid and difenoconazole reduced the fertilization rate, and thiram and imidacloprid reduced chick survival (Lopez-Antia *et al*., 2013). Similar effects have been detected in free-living birds. Female black-legged kittiwakes with higher levels of various pesticides and polychlorinated biphenyls (PCBs) in their blood samples exhibited duller orange–red labile integuments (i.e. eye ring, gapes and tongue), and these ornaments are believed to reflect individual quality in both sexes also (Blévin *et al*., 2014).

## Industry-related compounds

Many kinds of industrial pollutants potentially affect the colouration of ornamental traits in vertebrates, but only three have been studied in this context, namely PCBs, phenols (particularly bisphenol A and octylphenol) and PAHs.

Polychlorinated biphenyls are stable, human-made organic compounds that were commonly used in electrical applications, hydraulic equipment, plasticizers, paints, plastics, rubber production and as an insulating agent (reviewed by Blocker and Ophir, 2013). Production of PCBs ceased in 1972 (Dunlap, 1981) after their long-lasting toxic effects were realized, but the compounds persist in the environment and are capable of bio-accumulating with trophic position in lipid tissues. The PCBs are well known to disrupt endocrine function by agonizing and antagonizing natural oestrogens (Colborn *et al*., 1993) and negatively affecting thyroid function (Boas *et al*., 2006).

Similar endocrine-disrupting and oestrogenic effects occur from exposure to bisphenol A and octylphenol (Bonefeld-Jørgensen *et al*., 2007). These industrial pollutants typically reach wildlife through discharge to water courses, often via sewage treatment effluent (Kolpin *et al*., 2002). Despite its detrimental effects, production of bisphenol A is increasing because of its desirable commercial qualities for the manufacture of polycarbonate, epoxy and polyester resins (Crain *et al*., 2007). Octylphenol is a degradation byproduct of chemicals used in the manufacture of detergents and in agricultural and industrial products (Ying *et al*., 2002).

A third class of industrial pollutants that has been studied in the context of colouration is PAHs, which are the most toxic components of liquid petroleum products, but also occur as airborne pollutants (Eisler, 1987). Airborne PAHs typically result from combustion involving both anthropogenic sources (e.g. motor vehicles and many industrial processes) and natural ones (e.g. forest and prairie fires). Polycyclic aromatic hydrocarbons can reach aquatic environments via condensation in the atmosphere as well as via discharge of liquid waste through domestic and industrial sewage effluents, surface runoff from land, and spillage of petroleum products into water bodies (reviewed by Eisler, 1987). The acute toxicity of PAHs is mainly attributed to the oxidative stress they generate in exposed organisms, which adaptively activate their antioxidant system in order to survive in polluted environments (Pérez *et al*., 2010).

Among the few studies that have addressed the negative effects of PCBs on vertebrate colouration, only one examined melanic pigmentation. McCarty and Secord (2000) showed that subadult female tree swallows breeding along a river with high levels of PCB pollution displayed the blue–green iridescent plumage that is characteristic of adult birds of both sexes, rather than the brown upperparts that usually characterize subadult females. They speculated that the early expression of adult traits was caused by the oestrogenic properties of PCBs (McCarty and Secord, 2000). If delayed plumage maturation in females of this species is an adaptation to signal subordinate status, thereby reducing conspecific aggression (Coady and Dawson, 2013), earlier development of adult plumage may disadvantage these birds.

Surprisingly few studies have addressed the effects of PCBs on carotenoid-based colouration in fish and birds, given the enormous amount of literature that addresses their effects on more conventional physiological metrics (e.g. Eisler, 1987; Colborn *et al*., 1993; Flint *et al*., 2012), but they suggest consistently negative effects. For example, breeding male American kestrels that were exposed to a mix of PCB Aroclors lost brightness of the facial skin with yellow–orange pigments, which otherwise signals individual quality (Bortolotti *et al*., 2003). Likewise, black-legged kittiwakes showed dampening of the orange–red colouration in their faces with increasing concentrations of pesticides and PCBs in their blood (Blévin *et al*., 2014).

As a class of industrial chemicals, the effects of phenols on colouration have been studied only in fish. Guppies exposed to octylphenol produced fewer offspring, while displaying orange spots that were smaller (owing to inhibited growth of the spot) and less brightly coloured (Toft and Baatrup, 2001). Interestingly, cessation of exposure caused the colour but not size of the orange spots to recover, which might reflect functional differences in information content of the two colour signals. A similar effect occurred when male red shiners were exposed to the phenol bisphenol A for a period as short as 14 days, wherein there was a loss in the intensity of red breeding colouration in heads and fins and of blue iridescence in bodies (Ward and Blum, 2012). As an additional consequence of these colour changes, both sexes were less able to discriminate between conspecific and heterospecific partners during mate choice trials (Ward and Blum, 2012).

The effects of PAHs have been extensively studied in birds (Eisler, 1987), but their effects on ornament colouration have been explored only recently and in relation to the Prestige oil spill (González *et al*., 2006). Researchers working with yellow-legged gulls breeding in colonies located in the pathway of the spill focused on the carotenoid-based red bill spot, which is exhibited by both sexes and appears to reflect reliably the antioxidant status and capacity for parental investment of the bearer (Pérez *et al*., 2008; Morales *et al*., 2009). They found that the size of the red spot was negatively correlated with blood-based measures of aspartate aminotransferase, an enzyme that is commonly used as an indicator of hepatic damage in birds (Pérez *et al*., 2009). Additionally, gulls that were experimentally fed heavy fuel oil from the same spill had higher concentrations of PAHs, vitamin E and carotenoids in blood plasma, suggesting that the latter two had been mobilized for antioxidant defenses to cause the observed reductions in the size of the red bill spot (Pérez *et al*., 2010). Like the studies examining orange spots in guppies after exposure to pesticides (above), the work after the Prestige spill suggests that: (i) sexually selected, carotenoid-based traits respond very rapidly to environmental pollutants; and (ii) these traits might be particularly suitable as indicators after acute events.

## Metals

Several heavy metals, metalloids and trace elements are natural constituents of the Earth's crust, but they can also accumulate as a function of anthropogenic activity to become persistent environmental contaminants (Freedman, 2001). As metals cannot usually be degraded or destroyed, organisms that are exposed to metals via inhalation, absorption and ingestion often experience cumulative effects via one or both of bioaccumulation over time and biomagnification over trophic levels (Duruibe *et al*., 2007). Some elements, such as lead, mercury and arsenic, are toxic even at very low concentrations, but others, such as copper, selenium, iron and zinc, are essential to many biological processes and become toxic only at elevated concentrations (Ercal *et al*., 2001). Exposure to detrimental concentrations of either type can cause a cascade of effects that include decreased immune responsiveness (Kakuschke and Prange, 2007), increased oxidative stress (Valko *et al*., 2005) and, ultimately, lower survival or reproductive performance (Dauwe *et al*., 2004). The effects of metals on ornamental traits have been demonstrated for both melanins and carotenoids, but the direction of these effects differs.

For melanins, exposure to pollution from a lead smelter that included cadmium, zinc and copper resulted in larger eumelanic (black) breast stripes in great tits (Dauwe and Eens, 2008). This effect was surprising because larger stripes are preferred by females during mate choice (Norris, 1990). A similar positive trend was apparent in the brighter melanin-based plumage of belted kingfishers nesting close to a mercury-contaminated river and with higher levels of mercury in feathers and blood (White and Cristol, 2014). Like pesticides, metals seem to increase the expression of eumelanin-based ornamental traits, potentially disrupting the honesty of information they convey about bearer condition.

Also aligning with the evidence on ornamental traits for pesticides, metals appear to have a dampening effect on carotenoid-based ornaments. In great tits, pollution from metal smelters has been robustly demonstrated to reduce the carotenoid-based, yellow colouration in the breast feathers of both nestlings and adults to signal a loss in individual quality and condition (Eeva *et al*., 1998; Dauwe and Eens, 2008; Geens *et al*., 2009). The authors of these correlative studies emphasized different proximate effects of metal pollution on ornaments, which could include reduced access to carotenoid sources (Eeva *et al*., 1998), metal-induced oxidative stress (Geens *et al*., 2009), or both effects (Dauwe and Eens, 2008). It is already known that metal pollution reduces carotenoid synthesis in plants (Rai *et al*., 2005) which, in turn, could also reduce carotenoid concentrations in herbivores (e.g. caterpillars; Isaksson and Andersson, 2007). Whether they are predators or prey, individuals living in polluted sites appear generally to have reduced access to carotenoids, which may increase levels of oxidative stress to reduce condition in organisms with, as well as those without, carotenoid-based ornaments.

## Limitations to the use of colouration as an indicator of exposure to pollution

Several factors that we have not much addressed in our review potentially limit the use of sexually selected ornamental traits as indicators of exposure to pollution or other stressors. Most importantly, there must be visible and meaningful variation in ornaments among individuals that experience different environmental conditions over tractable scales of space and/or time. Nonetheless, the importance and meaning of traits based on a given kind of pigment can by highly variable between species. For example, in great tits (*Parus major*), the melanin-based breast stripe is condition dependent, whereas in American goldfinches (*Carduelis tristis*), the melanin-based black cap is not (McGraw and Hill, 2000; Fitze and Richner, 2002). Furthermore, there could even be variation in trait information content within a species. For example, in common yellowthroats (*Geothlypis trichas*), the melanin-based mask appears to be the target of sexual selection in one population, whereas the carotenoid-based bib conveys greater selective advantage in another population (Dunn *et al*., 2010). These examples demonstrate the need to examine, rather than assume, what information is conveyed by ornaments in a species- and even population-specific way before studying anthropogenic effects on those traits. This will be challenging because the rapidity of anthropogenic changes to environments may often exert strong selection pressures of their own (e.g. Candolin *et al*., 2007) that constrain, nullify or otherwise change the information contained in the variation that remains visible as the colour of ornaments.

Another important challenge for the use of ornaments as indicators of environmental pollutants will be to unravel the separate effects on multiple traits and their associated impacts on individual quality and fitness. Many species exhibit more than one ornamental trait that may function as redundancy (e.g. back-up signals; Møller and Pomiankowski, 1993), as multiple, or reinforcing, messages (Johnstone, 1996; Candolin, 2003) or even as evidence of trade-offs with each other (Andersson *et al*., 2002). Our review of the currently available literature suggests that ornaments coloured by different pigments may respond to a given pollutant in opposite directions [e.g. red-legged partridge (Alonso-Alvarez and Galván, 2011) and great tit (Dauwe and Eens, 2008)], but also that different ornaments formed with the same pigment respond in a similar manner (e.g. various carotenoid-based ornaments in the amarillo fish; Arellano-Aguilar and Macías Garcia, 2008).

A third challenge for the use of ornamental traits as indicators of pollution exposure is the inability of humans to see ornaments as other species might. A well-known example of this limitation is provided by the tetrachromatic vision of both birds (reviewed by Cuthill, 2006) and fish (Bowmaker, 1990), which affords detection of the ultraviolet portion of the colour spectrum. Ultraviolet colouration is already known to be used in mate choice and to reflect the condition and quality of the bearer (reviewed by Prum, 2006), and could provide another indicator of pollution exposure if it could be measured accurately. Although conventional digital cameras can be fitted with ultraviolet sensors (e.g. Lifepixel, 2016), their use in the field has been restricted thus far to tame and accessible species (e.g. Meyer-Rochow and Shimoyama, 2008). Even the melanic and carotenoid-based traits we measure more confidently could look very different to other species (reviewed by Cuthill, 2006).

## Recommendations for future research

Despite the limitations we acknowledge above, we see enormous potential for using ornamental traits that resulted from sexual selection to signal present-day exposure to anthropogenic pollutants. We suggest that in order to achieve this potential we will require the following: (i) the study and establishment of baselines of trait colour in the populations of interest; (ii) conducting broad-scale colour monitoring along different gradients of exposure to pollution; and (iii) an expansion in the existing techniques used to measure colouration, which are not much less invasive than the physiological metrics they might replace. Typically, quantification of colour still involves the capture and extensive handling of individuals, and those requirements potentially exclude many of the species that are most threatened by anthropogenic pollutants.

The first recommendation, the establishment of colour baselines, is necessary because of the enormous variability in the responses of ornamental traits to the wide variety of anthropogenic pollutants. Until more is known about how trait responses can be generalized among traits, species, pollutants, locations, etc., baseline metrics of trait colouration should be limited to small temporal and spatial scales with comparable environments. For example, bird feathers might be measured annually for a set of species in relation to standardized moult or breeding schedules and within a designated area. Once appropriate baselines are established, more robust comparisons could occur over space, time and anthropogenic conditions. Establishment of such baseline levels could create opportunities to assess predicted effects in such changes as land use practices, water treatment standards, industrial development and even the cumulative effects of adjacent human populations.

A second recommendation that could complement the development of the aforementioned baselines, is a broad-scale monitoring of ornamental colour. For this, the colour of ornamental traits of many species could be measured along known gradients of exposure to pollution to obtain a general picture of the sensitivity of such traits. This could be carried out repeatedly along several similar gradients to generalize the conclusions and along gradients of different sources of pollution. In addition to this, researchers or managers already monitoring populations and capturing individuals for other purposes could take colour measurements and/or photographs to expand our knowledge on colour variation in wild populations.

Given that the difficulty and invasiveness of capturing and taking samples from wild animals is a core motivator for our review paper, we see a primary research need as the development of remote sensing tools for measuring the colouration of ornamental traits. By remote sensing, we mean the use of cameras that can collect multispectral imagery while operating autonomously, in a similar manner to the way wildlife cameras are increasingly used for animal detection and surveillance (Turner *et al*., 2003; Swann *et al*., 2004; Bolton *et al*., 2007) and to the way vegetation type and phenological stage have long been assessed via multispectral sensors contained in satellites or aeroplanes. Already it is possible to combine these techniques via multispectral sensors in unmanned vehicles that can operate in each of air, land and water as media (reviewed by Linchant *et al*., 2015). The revolutionary potential of these vehicles has already been appreciated in many other conservation domains, and more applications are revealed steadily (reviewed by Conservation Drones, 2016). So far, the use of these tools for monitoring wildlife populations has been restricted mostly to detecting the presence of individuals or specific behaviours (e.g. Swann *et al*., 2004; Claridge *et al*., 2005; Grenzdörffer, 2013), but they are already being used to monitor the health of plants through changes in colouration that include the infrared portion of the colour spectrum (Fletcher *et al*., 2001; Newete *et al*., 2014).

Even if drones can be brought rapidly to the service of quantifying ornament colour for conservationist purposes, a related challenge is to create methods for calibrating photographs taken outdoors in varying conditions. Just as it is important to standardize assessments of coloured ornaments based on human observation (Montgomerie, 2006) and digital photography (Stevens *et al*., 2007), it will be important to develop methods to standardize images collected remotely. Although the same principles apply, much greater variation in light intensity and colour balance will apply to remote imagery collected outdoors. Variation in distance to subjects, resolution and orientation will make it harder to quantify relatively simple traits, such as ornament size. Overcoming the challenge of standardization will not be a trivial task, and it is probably best approached for this purpose within multidisciplinary teams that could include engineers, computing scientists and others who have solved similar problems for other purposes.

Equally important to standardizing the appearance of sexually selected ornaments by remotely collected photographs will be to continue the excellent pioneering work others have done to relate ornament colouration to physiological condition (e.g. Hamilton and Zuk, 1982; Kodric-Brown, 1985) and then to relate both metrics to environmental pollutants (e.g. Hill *et al*., 2002; Table 1). Although we focused our review on birds and fish because they have received all the attention to date, other taxonomic groups may offer advantages for the extension of these metrics via remote sensing. For example, amphibians are well known to be highly sensitive to environmental pollutants (Gibbons *et al*., 2000). Both amphibians and reptiles are typically terrestrial, which may make study via hand-held sensors in the wild easier than it would be for either birds or fish. The subjects of this work are not restricted to vertebrates; any taxonomic group that exhibits colourful integument, such as insects and crustaceans, is potentially relevant to advancing a general understanding of the way colouration could signal the detrimental presence or effects of anthropogenic pollution. With such general knowledge, conservation practitioners might be able both to detect and to mitigate the effects of pollution long before they cause local population declines or extirpation.

A final target of future research is to identify generalizations from studies of sexually selected indicators and use them to predict which species, populations and individuals are most likely to adapt to rather than deteriorate from changing environments. One example of this proactive approach would be to target the closely related, sympatric species that are more likely to use colourful ornamental traits for premating reproductive barriers (Ritchie, 2007; Price, 2008). Without those barriers, hybridization and genetic introgression could cause reductions in offspring survival (Pryke and Griffith, 2009) or the loss of local adaptations (Bourret *et al*., 2011), ultimately contributing to extinctions (Rhymer and Simberloff, 1996). A well-known example of this sequence occurred with cichlid fishes in Lake Victoria. There, intensified deforestation and agricultural practices increased water turbidity and reduced colour perception by fish, which increased rates of hybridization to cause cascading losses of fish diversity (Seehausen *et al*., 1997).

Another proactive research target would be to determine the extent and implications of increased expression of melanic traits in individuals that have been exposed to pollution. The ability of melanin to bind with metal ions means that animals with melanic ornaments can eliminate excess metals, which are essential in small quantities but toxic in higher concentrations, by sequestering them in hair of feathers (McGraw, 2003). Recent work suggests that this mechanism can cause directional selection for more melanic phenotypes in polluted environments (Chatelain *et al*., 2014), but the reverse may also occur (Senar *et al*., 2014). If melanin-based sequestration of toxicants is widespread in animals, it could have big implications for changing patterns of biodiversity, especially in urban areas. For example, McKinney's (2002) categorization of bird species as urban avoiders, adapters and exploiters was originally based on correlations between life-history traits and abundance, but a systematic review might reveal that the same categories correlate with the prevalence of melanic traits. The intriguing relationships among metal pollution and melanins have been most explored in birds, but they deserve investigation in mammals, especially in invasive urban-adapting species that exhibit variation in both black and reddish colouration caused by melanins (e.g. coyotes, *Canis latrans*; Anderson *et al*., 2009). Clearly, much more research is needed to address how melanins interact with a variety of anthropogenic effects.

## Conclusions

Exponential growth in human populations and associated habitat degradation increasingly threaten the retention of biodiversity on Earth. Stemming this loss will require efficient conservation action that identifies the mechanisms of population decline, acts proactively to reduce the cost and increase the efficacy of mitigation, and operates holistically to generalize efforts across ecological units and scales. When these three things can be achieved, the results are heartening. For example, the identification of volatile chlorofluorocarbons in aerosol sprays as the cause of ozone depletion resulted in rapid regulatory change, widespread compliance, the recovery of an essential ecosystem service, and benefits to hundreds of species (Noakes, 1995).

Unfortunately, that critical first step—identifying the mechanism of population decline—is surprisingly difficult, especially when it involves anthropogenic pollutants with nebulous components, sources and effects. Millions of chemicals are produced annually and released into the environment (Postel, 1987), but only a small fraction of those are tested for their environmental impacts (Tolba, 1992). There is an enormous need to develop and apply more methods to monitor the health of wildlife exposed to new and existing anthropogenic pollutants, but current methods are invasive, sometimes lethal (e.g. Farombi *et al*., 2007), often limited to blood-based or reproductive parameters (e.g. Fernie *et al*., 2001) or are reliant on opportunistic observations of mortality (e.g. Piatt *et al*., 1990). Additionally, none of these methods is suitable for estimating effects of pollutants in wild but sensitive populations that inhabit locations that are remote, vulnerable to disturbance, difficult to access or endangered. Alas, those are precisely the species, populations and locations where such assessments are most needed to support proactive and effective conservation action!

In this review, we champion an idea presented by Hill (1995), more than 20 years ago, that the ornamental traits of many organisms could be used to fill this void, revealing the quality and condition of environments that are also occupied by many other species. Since then, many researchers have explored species- and trait-specific effects of particular pollutants to accumulate a substantial literature. We synthesized and reviewed that literature in relation to two types of pigments that are prevalent in ornaments in vertebrate classes, i.e. melanins and carotenoids. We also assessed their known interactions with several environmental pollutants. Despite the number of relevant studies, most of this literature describes laboratory or case studies and is usually specific to taxonomic groups or particular toxicological effects. These characteristics impede the emergence of generalizable mechanisms that could reveal wider conservation problems and suggest appropriate mitigations. Consequently, the potential for ornamental traits to comprise a versatile tool for conservation biology remains unmet. We see seminal roles for development of this theory using pollution as an anthropogenic effect, ornamented vertebrates as study subjects, and pigments as mechanistic intermediaries. These targets are illustrative because pollution often interferes with the biochemical creation and physiological maintenance of ornamental traits, which are prevalently expressed with pigments. Moreover, pigments are costly to synthesize or acquire, are fairly well understood in the contexts of biochemistry, physiology, toxicology and sexual selection, and should be readily visible to both human observers and their automated devices. With time, we expect that ornamental traits will become familiar tools for diagnosing and addressing a wide variety of anthropogenic effects, with tractable applications for hundreds of species and ecosystems worldwide.

We attempted to advance Hill's (1995) insight by reviewing that literature and using it to pose ideas for future research that could help generalize the use of this method, while acknowledging some important limitations. We hope this review encourages more researchers and wildlife managers to include measures of ornamental colour whenever possible and, especially, when it can easily be added to existing monitoring or research protocols. We see enormous potential for these traits to provide a much-needed, non-invasive tool for detecting subtle and early effects of pollution long before they can be seen as more catastrophic effects at the level of populations. Species that exhibit carotenoid-based ornaments appear to offer particular promise as highly responsive indicators that might reveal pollution exposure that is not easily detected, but actually occurs, in other species and even whole communities.

Despite the great potential we see in the utility of ornamental traits as environmental indicators, we also emphasize the importance of unravelling the specific physiological mechanisms of pollutants on colouration before generalizing their interpretations, particularly when anthropogenic stressors cause opposing or multiplicative effects. A striking contradiction is evident in the enhancing effects of both pesticides and heavy metals on eumelanic (dark) traits, with simultaneous negative effects on pheomelanic (lighter) and carotenoid-based traits that are undoubtedly detrimental for organisms.

More systematic work on the specific sources, mechanisms and mitigations of pollutant effects on sexually selected ornaments could provide conservation biologists with a suite of context-specific canaries for the diverse coal mines of anthropogenic effects. At the same time, such work may provide unanticipated insights relevant to more basic questions in physiology, ecology and evolution.

## References

[cow028C1] Abdollahi M, Ranjbar A, Shadnia S, Nikfar S, Rezaiee A (2004) Pesticides and oxidative stress: a review. Med Sci Monit 10: RA141–RA147. 15173684

[cow028C2] Alonso-Alvarez C, Galván I (2011) Free radical exposure creates paler carotenoid-based ornaments: a possible interaction in the expression of black and red traits. PLoS ONE 6: e19403. 21556328 10.1371/journal.pone.0019403 PMC3083443

[cow028C3] Anderson TM, Candille SI, Musiani M, Greco C, Stahler DR, Smith DW, Padhukasahasram B, Randi E, Leonard JA, Bustamante CD et al (2009) Molecular and evolutionary history of melanism in North American gray wolves. Science 323: 1339–1343. 19197024 10.1126/science.1165448 PMC2903542

[cow028C4] Andersson S, Pryke SR, Örnborg J, Lawes MJ, Andersson M (2002) Multiple receivers, multiple ornaments, and a trade‐off between agonistic and epigamic signaling in a widowbird. Am Nat 160: 683–691. 18707516 10.1086/342817

[cow028C5] Arellano-Aguilar O, Macías Garcia C (2008) Exposure to pesticides impairs the expression of fish ornaments reducing the availability of attractive males. Proc Biol Sci 275: 1343–1350. 18348963 10.1098/rspb.2008.0163 PMC2602681

[cow028C7] Baatrup E, Junge M (2001) Antiandrogenic pesticides disrupt sexual characteristics in the adult male guppy (*Poecilia reticulata*). Environ Health Perspect 109: 1063–1070. 11675272 10.1289/ehp.011091063 PMC1242084

[cow028C8] Badyaev AV, Hill GE (2000) Evolution of sexual dichromatism: contribution of carotenoid‐ versus melanin‐based coloration. Biol J Linn Soc 69: 153–172.

[cow028C9] Baruch-Mordo S, Evans JS, Severson JP, Naugle DE, Maestas JD, Kiesecker JM, Falkowski MJ, Hagen CA, Reese KP (2013) Saving sage-grouse from the trees: a proactive solution to reducing a key threat to a candidate species. Biol Conserv 167: 233–241.

[cow028C10] Bickham JW, Sandhu S, Hebert PD, Chikhi L, Athwal R (2000) Effects of chemical contaminants on genetic diversity in natural populations: implications for biomonitoring and ecotoxicology. Mutat Res 463: 33–51. 10838208 10.1016/s1383-5742(00)00004-1

[cow028C11] Blévin P, Tartu S, Angelier F, Leclaire S, Bustnes JO, Moe B, Herzke D, Gabrielsen GW, Chastel O (2014) Integument colouration in relation to persistent organic pollutants and body condition in Arctic breeding black-legged kittiwakes (*Rissa tridactyla*). Sci Total Environ 470: 248–254. 24140695 10.1016/j.scitotenv.2013.09.049

[cow028C12] Blocker TD, Ophir AG (2013) Cryptic confounding compounds: a brief consideration of the influences of anthropogenic contaminants on courtship and mating behavior. Acta Ethol 16: 105–125. 10.1007/s10211-012-0137-x PMC3827776 24244068

[cow028C13] Boas M, Feldt-Rasmussen U, Skakkebæk NE, Main KM (2006) Environmental chemicals and thyroid function. Eur J Endocrinol 154: 599–611. 16645005 10.1530/eje.1.02128

[cow028C14] Bókony V, Garamszegi LZ, Hirschenhauser K, Liker A (2008) Testosterone and melanin-based black plumage coloration: a comparative study. Behav Ecol Sociobiol 62: 1229–1238.

[cow028C15] Bolton M, Butcher N, Sharpe F, Stevens D, Fisher G (2007) Remote monitoring of nests using digital camera technology. J Field Ornithol 78: 213–220.

[cow028C16] Bonefeld-Jørgensen EC, Long M, Hofmeister MV, Vinggaard AM (2007) Endocrine-disrupting potential of bisphenol A, bisphenol A dimethacrylate, 4-*n*-nonylphenol, and 4-*n*-octylphenol *in vitro*: new data and a brief review. Environ Health Perspect 115: 69–76. 18174953 10.1289/ehp.9368 PMC2174402

[cow028C18] Bortolotti GR, Fernie KJ, Smits JE (2003) Carotenoid concentration and coloration of American kestrels (*Falco sparverius*) disrupted by experimental exposure to PCBs. Funct Ecol 17: 651–657.

[cow028C19] Bortolotti GR, Blas J, Negro JJ, Tella JL (2006) A complex plumage pattern as an honest social signal. Anim Behav 72: 423–430.

[cow028C21] Bourret V, O'Reilly P, Carr J, Berg P, Bernatchez L (2011) Temporal change in genetic integrity suggests loss of local adaptation in a wild Atlantic salmon (*Salmo salar*) population following introgression by farmed escapees. Heredity (Edinb) 106: 500–510. 21224876 10.1038/hdy.2010.165 PMC3131974

[cow028C22] Bowmaker JK (1990) Visual pigments of fishes In Douglas RH, Djamgoz MBA, eds, The Visual System of Fish. Chapman and Hall, London, pp 81–107.

[cow028C23] Buchanan KL (2000) Stress and the evolution of condition-dependent signals. Trends Ecol Evol 15: 156–160. 10717685 10.1016/s0169-5347(99)01812-1

[cow028C24] Buchholz R (2007) Behavioural biology: an effective and relevant conservation tool. Trends Ecol Evol 22: 401–407 17590477 10.1016/j.tree.2007.06.002

[cow028C25] Candolin U (2003) The use of multiple cues in mate choice. Biol Rev Camb Philos Soc 78: 575–595. 14700392 10.1017/s1464793103006158

[cow028C26] Candolin U, Salesto T, Evers M (2007) Changed environmental conditions weaken sexual selection in sticklebacks. J Evol Biol 20: 233–239. 17210016 10.1111/j.1420-9101.2006.01207.x

[cow028C27] Caro T, Sherman PW (2013) Eighteen reasons animal behaviourists avoid involvement in conservation. Anim Behav 85: 305–312.

[cow028C28] Caughley G (1994) Directions in conservation biology. J Anim Ecol 63: 215–244.

[cow028C29] Chatelain M, Gasparini J, Jacquin L, Frantz A (2014) The adaptive function of melanin-based plumage coloration to trace metals. Biol Lett 10: 20140164. 24671830 10.1098/rsbl.2014.0164 PMC3982444

[cow028C31] Claridge AW, Mifsud G, Dawson J, Saxon MJ (2005) Use of infrared digital cameras to investigate aspects of the social behaviour of cryptic species. Wildlife Res 31: 645–665.

[cow028C32] Clout MN, Elliott GP, Robertson BC (2002) Effects of supplementary feeding on the offspring sex ratio of kakapo: a dilemma for the conservation of a polygynous parrot. Biol Conserv 107: 13–18.

[cow028C33] Coady CD, Dawson RD (2013) Subadult plumage color of female tree swallows (*Tachycineta bicolor*) reduces conspecific aggression during the breeding season. Wilson J Ornithol 125: 348–357.

[cow028C34] Colborn T, vom Saal FS, Soto AM (1993) Developmental effects of endocrine-disrupting chemicals in wildlife and humans. Environ Health Perspect 101: 378–384. 8080506 10.1289/ehp.93101378 PMC1519860

[cow028C35] Conservation Drones (2016) http://www.conservationdrones.org. (last accessed 7 June 2016).

[cow028C36] Cotton S, Fowler K, Pomiankowski A (2004) Do sexual ornaments demonstrate heightened condition-dependent expression as predicted by the handicap hypothesis. Proc Biol Sci 271: 771–783. 15255094 10.1098/rspb.2004.2688 PMC1691662

[cow028C37] Crain DA, Eriksen M, Iguchi T, Jobling S, Laufer H, LeBlanc GA, Guillette LJ (2007) An ecological assessment of bisphenol-A: evidence from comparative biology. Reprod Toxicol 24: 225–239. 17604601 10.1016/j.reprotox.2007.05.008

[cow028C38] Cuthill IC (2006) Color perception In Hill GE, McGraw KJ, eds, Bird Coloration, Vol I, Mechanisms and Measurements. Harvard University Press, Cambridge, MA, pp 3–40.

[cow028C39] Darwin C (1871) The Descent of Man, and Selection in Relation to Sex. John Murray, London.

[cow028C40] Daughton CG, Ternes TA (1999) Pharmaceuticals and personal care products in the environment: agents of subtle change. Environ Health Perspect 107: 907–938. 10592150 10.1289/ehp.99107s6907 PMC1566206

[cow028C41] Dauwe T, Eens M (2008) Melanin- and carotenoid-dependent signals of great tits (*Parus major*) relate differently to metal pollution. Naturwissenschaften 95: 969–973. 18506415 10.1007/s00114-008-0400-1

[cow028C42] Dauwe T, Janssens E, Kempenaers B, Eens M (2004) The effect of heavy metal exposure on egg size, eggshell thickness and the number of spermatozoa in blue tit *Parus caeruleus* eggs. Environ Pollut 129: 125–129. 14749076 10.1016/j.envpol.2003.09.028

[cow028C43] Dunlap T (1981) DDT: Scientists, Citizens and Public Policy. Princeton University Press, Princeton, NY.

[cow028C44] Dunn PO, Garvin JC, Whittingham LA, Freeman‐Gallant CR, Hasselquist D (2010) Carotenoid and melanin‐based ornaments signal similar aspects of male quality in two populations of the common yellowthroat. Funct Ecol 24:149–158.

[cow028C45] Duruibe J, Ogwuegbu M, Egwurugwu J (2007) Heavy metal pollution and human biotoxic effects. Int J Phys Sci 2: 112–118.

[cow028C46] Eeva T, Lehikoinen E, Rönkä M (1998) Air pollution fades the plumage of the great tit. Funct Ecol 12: 607–612.

[cow028C47] Eisler R (1987) Polycyclic aromatic hydrocarbon hazards to fish, wildlife, and invertebrates: a synoptic review. Biological Report 85 (1.11), US Fish and Wildlife Service, Laurel, MD.

[cow028C48] Ercal N, Gurer-Orhan H, Aykin-Burns N (2001) Toxic metals and oxidative stress part I: mechanisms involved in metal-induced oxidative damage. Curr Top Med Chem 1: 529–539. 11895129 10.2174/1568026013394831

[cow028C49] Faivre B, Grégoire A, Préault M, Cézilly F, Sorci G (2003) Immune activation rapidly mirrored in a secondary sexual trait. Science 300: 103. 12677062 10.1126/science.1081802

[cow028C50] Fargallo JA, Laaksonen T, Korpimäki E, Wakamatsu K (2007). A melanin-based trait reflects environmental growth conditions of nestling male Eurasian kestrels. Evol Ecol 21:157–171.

[cow028C51] Farombi E, Adelowo O, Ajimoko Y (2007) Biomarkers of oxidative stress and heavy metal levels as indicators of environmental pollution in African cat fish (*Clarias gariepinus*) from Nigeria Ogun River. Int J Environ Res Public Health 4: 158–165. 17617680 10.3390/ijerph2007040011 PMC3728582

[cow028C52] Fernie KJ, Smits JE, Bortolotti GR, Bird DM (2001) Reproduction success of American kestrels exposed to dietary polychlorinated biphenyls. Environ Toxicol Chem 20: 776–781. 11345453

[cow028C54] Fitze PS, Richner H (2002) Differential effects of a parasite on ornamental structures based on melanins and carotenoids. Behav Ecol 13: 401–407.

[cow028C55] Fletcher RS, Skaria M, Escobar DE, Everitt JH (2001) Field spectra and airborne digital imagery for detecting phytophthora foot rot infections in citrus trees. HortScience 36: 94–97.

[cow028C56] Flint S, Markle T, Thompson S, Wallace E (2012) Bisphenol A exposure, effects, and policy: a wildlife perspective. J Environ Manage 104: 19–34. 22481365 10.1016/j.jenvman.2012.03.021

[cow028C57] Freedman B (2001) Environmental Science: A Canadian Perspective. Prentice Hall, Toronto, Ontario, Canada.

[cow028C58] Galván I, Alonso-Alvarez C (2008) An intracellular antioxidant determines the expression of a melanin-based signal in a bird. PLoS ONE 3: e3335. 18833330 10.1371/journal.pone.0003335 PMC2556083

[cow028C59] Galván I, Alonso-Alvarez C (2009) The expression of melanin-based plumage is separately modulated by exogenous oxidative stress and a melanocortin. Proc Biol Sci 276: 3089–3097. 19520801 10.1098/rspb.2009.0774 PMC2817136

[cow028C61] Geens A, Dauwe T, Eens M (2009) Does anthropogenic metal pollution affect carotenoid colouration, antioxidative capacity and physiological condition of great tits (*Parus major*). Comp Biochem Physiol C Toxicol Pharmacol 150: 155–163. 19394439 10.1016/j.cbpc.2009.04.007

[cow028C62] Gibbons JW, Scott DE, Ryan TJ, Buhlmann KA, Tuberville TD, Metts BS, Greene JL, Mills T, Leiden Y, Poppy S et al (2000) The global decline of reptiles, déjà vu amphibians. BioScience 50: 653–666.

[cow028C63] González J, Viñas L, Franco M, Fumega J, Soriano J, Grueiro G, Muniategui S, López-Mahía P, Prada D, Bayona J et al (2006) Spatial and temporal distribution of dissolved/dispersed aromatic hydrocarbons in seawater in the area affected by the *Prestige* oil spill. Mar Pollut Bull 53: 250–359. 16274705 10.1016/j.marpolbul.2005.09.039

[cow028C64] Goutte A, Barbraud C, Meillère A, Carravieri A, Bustamante P, Labadie P, Budzinski H, Delord K, Cherel Y, Weimerskirch H et al (2014) Demographic consequences of heavy metals and persistent organic pollutants in a vulnerable long-lived bird, the wandering albatross. Proc Biol Sci 281: 20133313. 24920477 10.1098/rspb.2013.3313 PMC4071534

[cow028C65] Grafen A (1990) Biological signals as handicaps. J Theor Biol 144: 517–546. 2402153 10.1016/s0022-5193(05)80088-8

[cow028C66] Grenzdörffer GJ (2013) UAS-based automatic bird count of a common gull colony. Int Arch Photogramm Remote Sens Spat Inf Sci XL-1/W2: 169–174.

[cow028C67] Griffith SC, Owens IPF, Burke T (1999) Environmental determination of a sexually selected trait. Nature 400: 358–360.

[cow028C68] Guindre‐Parker S, Love OP (2014) Revisiting the condition‐dependence of melanin‐based plumage. J Avian Biol 45: 29–33.

[cow028C69] Halliwell B, Gutteridge J (2007) Free Radicals in Biology and Medicine, Ed 4 Oxford University Press, Oxford.

[cow028C70] Hamilton WD, Zuk M (1982) Heritable true fitness and bright birds: a role for parasites. Science 218: 384–387. 7123238 10.1126/science.7123238

[cow028C71] Hartley RC, Kennedy MW (2004) Are carotenoids a red herring in sexual display. Trends Ecol Evol 19: 353–354. 16701285 10.1016/j.tree.2004.04.002

[cow028C72] Hill GE (1995) Ornamental traits as indicators of environmental health: condition-dependent display traits hold promise as potent biomonitors. BioScience 45: 25–31.

[cow028C73] Hill GE, McGraw KJ (2006a) Bird Coloration, Vol I, Mechanisms and Measurements. Harvard University Press, Cambridge, MA.

[cow028C74] Hill GE, McGraw KJ (2006b) Bird Coloration, Vol II, Function and Evolution. Harvard University Press, Cambridge, MA.

[cow028C75] Hill GE, Inouye CY, Montgomerie R (2002) Dietary carotenoids predict plumage coloration in wild house finches. Proc Biol Sci 269: 1119–1124. 12061954 10.1098/rspb.2002.1980 PMC1691014

[cow028C78] Isaksson C (2010) Pollution and its impact on wild animals: a meta-analysis on oxidative stress. EcoHealth 7: 342–350. 20865439 10.1007/s10393-010-0345-7

[cow028C79] Isaksson C, Andersson S (2007) Carotenoid diet and nestling provisioning in urban and rural great tits *Parus major*. J Avian Biol 38: 564–572.

[cow028C80] Jawor JM, Breitwisch R (2003) Melanin ornaments, honesty, and sexual selection. Auk 120: 249–265.

[cow028C82] Johnstone RA (1996) Multiple displays in animal communication: ‘backup signals’ and ‘multiple messages’. Philos Trans R Soc Lond B Biol Sci 351: 329–338.

[cow028C83] Kakuschke A, Prange A (2007) The influence of metal pollution on the immune system: a potential stressor for marine mammals in the North Sea. Int J Comp Psychol 20: 179–193.

[cow028C84] Kidd KA, Blanchfield PJ, Mills KH, Palace VP, Evans RE, Lazorchak JM, Flick RW (2007) Collapse of a fish population after exposure to a synthetic estrogen. Proc Natl Acad Sci USA 104: 8897–8901. 17517636 10.1073/pnas.0609568104 PMC1874224

[cow028C85] Kodric-Brown A (1985) Female preference and sexual selection for male coloration in the guppy (*Poecilia reticulata*). Behav Ecol Sociobiol 17: 199–205.

[cow028C86] Kodric-Brown A, Nicoletto PF (2001) Female choice in the guppy (*Poecilia reticulata*): the interaction between male color and display. Behav Ecol Sociobiol 50: 346–351.

[cow028C88] Kolpin DW, Furlong ET, Meyer MT, Thurman EM, Zaugg SD, Barber LB, Buxton HT (2002) Pharmaceuticals, hormones, and other organic wastewater contaminants in US streams, 1999–2000: a national reconnaissance. Environ Sci Technol 36: 1202–1211. 11944670 10.1021/es011055j

[cow028C89] Kristensen T, Baatrup E, Bayley M (2005) 17α-Ethinylestradiol reduces the competitive reproductive fitness of the male guppy (*Poecilia reticulata*). Biol Reprod 72: 150–156. 15355877 10.1095/biolreprod.104.033001

[cow028C91] Larsen MG, Hansen KB, Henriksen PG, Baatrup E (2008) Male zebrafish (*Danio rerio*) courtship behaviour resists the feminising effects of 17α-ethinyloestradiol—morphological sexual characteristics do not. Aquat Toxicol 87: 234–244. 18359522 10.1016/j.aquatox.2008.02.003

[cow028C92] Lifepixel (2016) http://www.lifepixel.com/?s=uv+conversion (last accessed 7 June 2016).

[cow028C93] Lin JY, Fisher DE (2007) Melanocyte biology and skin pigmentation. Nature 445: 843–850. 17314970 10.1038/nature05660

[cow028C94] Linchant J, Lisein J, Semeki J, Lejeune P, Vermeulen C (2015) Are unmanned aircraft systems (UASs) the future of wildlife monitoring? A review of accomplishments and challenges. Mamm Rev 45: 239–252.

[cow028C95] Lopez-Antia A, Ortiz-Santaliestra ME, Mougeot F, Mateo R (2013) Experimental exposure of red-legged partridges (*Alectoris rufa*) to seeds coated with imidacloprid, thiram and difenoconazole. Ecotoxicology 22: 125–138. 23111803 10.1007/s10646-012-1009-x

[cow028C96] Lozano GA (1994) Carotenoids, parasites, and sexual selection. Oikos 70: 309–311.

[cow028C97] McCarty JP, Secord AL (2000) Possible effects of PCB contamination on female plumage color and reproductive success in Hudson River Tree Swallows. Auk 117: 987–995.

[cow028C98] McGraw KJ (2003) Melanins, metals, and mate quality. Oikos 1: 402–406.

[cow028C99] McGraw KJ (2005) The antioxidant function of many animal pigments: are there consistent health benefits of sexually selected colourants. Anim Behav 69: 757–764.

[cow028C100] McGraw KJ (2006a) Mechanisms of carotenoid-based coloration In Hill GE, McGraw KJ, eds, Bird Coloration, Vol I, Mechanisms and Measurements. Harvard University Press, Cambridge, MA, pp 177–242.

[cow028C101] McGraw KJ (2006b) Mechanisms of melanin-based coloration In Hill GE, McGraw KJ, eds, Bird Coloration, Vol I, Mechanisms and Measurements. Harvard University Press, Cambridge, MA, pp 243–294.

[cow028C102] McGraw KJ (2008) An update on the honesty of melanin‐based color signals in birds. Pigment Cell Melanoma Res 21: 133–138. 18426406 10.1111/j.1755-148X.2008.00454.x

[cow028C103] McGraw KJ, Hill GE (2000) Differential effects of endoparasitism on the expression of carotenoid- and melanin-based ornamental coloration. Proc Biol Sci 267: 1525–1531. 11007328 10.1098/rspb.2000.1174 PMC1690705

[cow028C104] McGree MM, Winkelman DL, Vieira NK, Vajda AM (2010) Reproductive failure of the red shiner (*Cyprinella lutrensis*) after exposure to an exogenous estrogen. Can J Fish Aquat Sci 67: 1730–1743.

[cow028C105] Macías Garcia C (1991) Sexual behaviour and trade-offs in the viviparous fish *Girardinichthys multiradiatus* PhD thesis, University of East Anglia, Norwich, UK.

[cow028C106] McKinney ML (2002) Urbanization, biodiversity, and conservation. BioScience 52: 883–890.

[cow028C107] Martínez-Abraín A, Velando A, Oro D, Genovart M, Gerique C, Bartolomé MA, Villuendas E, Sarzo B (2006) Sex-specific mortality of European shags after the Prestige oil spill: demographic implications for the recovery of colonies. Mar Ecol Prog Ser 318: 271–276.

[cow028C109] Meyer-Rochow VB, Shimoyama A (2008) UV-reflecting and absorbing body regions in gentoo and king penguin: can they really be used by the penguins as signals for conspecific recognition. Polar Biol 31: 557–560.

[cow028C111] Møller AP, Pomiankowski A (1993) Why have birds got multiple sexual ornaments. Behav Ecol Sociobiol 32: 167–176.

[cow028C112] Monaghan P (2008) Early growth conditions, phenotypic development and environmental change. Philos Trans R Soc Lond B Biol Sci 363: 1635–1645. 18048301 10.1098/rstb.2007.0011 PMC2606729

[cow028C113] Monaghan P, Metcalfe NB, Torres R (2009) Oxidative stress as a mediator of life history trade‐offs: mechanisms, measurements and interpretation. Ecol Lett 12: 75–92. 19016828 10.1111/j.1461-0248.2008.01258.x

[cow028C114] Montgomerie R (2006) Analyzing colors In Hill GE, McGraw KJ, eds, Bird Coloration, Vol I, Mechanisms and Measurements. Harvard University Press, Cambridge, MA, pp 90–147.

[cow028C116] Morales J, Alonso-Álvarez C, Pérez C, Torres R, Serafino E, Velando A (2009) Families on the spot: sexual signals influence parent–offspring interactions. Proc Biol Sci 276: 2477–2483. 19364749 10.1098/rspb.2008.1942 PMC2690456

[cow028C117] Mougeot F, Pérez‐Rodríguez L, Sumozas N, Terraube J (2009) Parasites, condition, immune responsiveness and carotenoid‐based ornamentation in male red‐legged partridge *Alectoris rufa*. J Avian Biol 40: 67–74.

[cow028C118] Naguib M, Nemitz A (2007) Living with the past: nutritional stress in juvenile males has immediate effects on their plumage ornaments and on adult attractiveness in zebra finches. PLoS ONE 2: e901. 17878936 10.1371/journal.pone.0000901 PMC1975674

[cow028C119] Newete SW, Erasmus BF, Weiersbye IM, Cho MA, Byrne MJ (2014) Hyperspectral reflectance features of water hyacinth growing under feeding stresses of *Neochetina* spp. and different heavy metal pollutants. Int J Remote Sens 35: 799–817.

[cow028C120] Noakes TJ (1995) CFCs, their replacements, and the ozone layer. J Aerosol Med 8: S-3–S-7. 10150493 10.1089/jam.1995.8.suppl_1.s-3

[cow028C121] Norris KJ (1990) Female choice and the evolution of the conspicuous plumage coloration of monogamous male great tits. Behav Ecol Sociobiol 26: 129–138.

[cow028C124] Pérez C, Lores M, Velando A (2008) The availability of nonpigmentary antioxidant affects red coloration in gulls. Behav Ecol 19: 967–973.

[cow028C125] Pérez C, Munilla I, López-Alonso M, Velando A (2009) Sublethal effects on seabirds after the *Prestige* oil-spill are mirrored in sexual signals. Biol Lett 6: 33–35. 19726443 10.1098/rsbl.2009.0567 PMC2817249

[cow028C126] Pérez C, Lores M, Velando A (2010) Oil pollution increases plasma antioxidants but reduces coloration in a seabird. Oecologia 163: 875–884. 20532916 10.1007/s00442-010-1677-2

[cow028C127] Pérez-Rodríguez L (2009) Carotenoids in evolutionary ecology: re‐evaluating the antioxidant role. BioEssays 31: 1116–1126. 19705366 10.1002/bies.200900070

[cow028C128] Pérez-Rodríguez L, Viñuela J (2008) Carotenoid-based bill and eye ring coloration as honest signals of condition: an experimental test in the red-legged partridge (*Alectoris rufa*). Naturwissenschaften 95: 821–830. 18470503 10.1007/s00114-008-0389-5

[cow028C129] Piatt JF, Lensink CJ, Butler W, Kendziorek M, Nysewander DR (1990) Immediate impact of the ‘Exxon Valdez’ oil spill on marine birds. Auk 107: 387–397.

[cow028C130] Pimentel D, Acquay H, Biltonen M, Rice P, Silva M, Nelson J, Lipner V, Giordano S, Horowitz A, D'Amore M (1992) Environmental and economic costs of pesticide use. BioScience 42: 750–760.

[cow028C131] Plasman M, Reynoso V, Nicolás L, Torres R (2015) Multiple colour traits signal performance and immune response in the Dickerson's collared lizard *Crotaphytus dickersonae*. Behav Ecol Sociobiol 69: 765–775.

[cow028C132] Postel S (1987) Defusing the toxics threat: controlling pesticides and industrial waste. Worldwatch paper 79. Worldwatch Institute, Washington, DC.

[cow028C133] Price T (2008) Speciation in Birds. Roberts and Company, Greenwood Village, CO.

[cow028C134] Prum RO (2006) Anatomy, physics, and evolution of avian structural colors In Hill GE, McGraw KJ, eds, Bird Coloration, Vol I, Mechanisms and Measurements. Harvard University Press, Cambridge, MA, pp 295–353.

[cow028C135] Pryke SR, Griffith SC (2009) Postzygotic genetic incompatibility between sympatric color morphs. Evolution 63: 793–798. 19087185 10.1111/j.1558-5646.2008.00584.x

[cow028C136] Rai V, Khatoon S, Bisht S, Mehrotra S (2005) Effect of cadmium on growth, ultramorphology of leaf and secondary metabolites of *Phyllanthus amarus* Schum. and Thonn. Chemosphere 61: 1644–1650. 15992855 10.1016/j.chemosphere.2005.04.052

[cow028C137] Rhymer JM, Simberloff D (1996) Extinction by hybridization and introgression. Annu Rev Ecol Syst 27: 83–109.

[cow028C138] Ritchie MG (2007) Sexual selection and speciation. Annu Rev Ecol Evol Syst 38: 79–102.

[cow028C139] Ross PS, Vedder L, Timmerman H, Heisterkamp S, van Loveren H, Vos J, Reijnders P (1994) Impairment of immune function in harbor seals (*Phoca vitulina*) feeding on fish from polluted waters. Ambio 23: 155–159.

[cow028C141] Safran RJ, McGraw KJ (2004) Plumage coloration, not length or symmetry of tail-streamers, is a sexually selected trait in North American barn swallows. Behav Ecol 15: 455–461.

[cow028C142] Schiedt K (1989) New aspects of carotenoid metabolism in animals In Krinsky NI, Mathews-Roth MM, Taylor RF, eds, Carotenoids: Chemistry and Biology. Plenum Press, New York, pp 247–268.

[cow028C143] Seehausen O, Van Alphen JJ, Witte F (1997) Cichlid fish diversity threatened by eutrophication that curbs sexual selection. Science 277: 1808–1811.

[cow028C144] Senar JC, Conroy MJ, Quesada J, Mateos‐Gonzalez F (2014) Selection based on the size of the black tie of the great tit may be reversed in urban habitats. Ecol Evol 4: 2625–2632. 25077014 10.1002/ece3.999 PMC4113287

[cow028C146] Shenoy K (2012) Environmentally realistic exposure to the herbicide atrazine alters some sexually selected traits in male guppies. PLoS ONE 7: e30611. 22312428 10.1371/journal.pone.0030611 PMC3270011

[cow028C148] Stevens M, Páraga CA, Cuthill IC, Partridge JC, Troscianko TS (2007) Using digital photography to study animal coloration. Biol J Linn Soc 90: 211–237.

[cow028C149] Stutchbury B (2009) Silence of the Songbirds. Harper Collins, New York.

[cow028C150] Sutherland WJ (1998) The importance of behavioural studies in conservation biology. Anim Behav 56: 801–809. 9790690 10.1006/anbe.1998.0896

[cow028C151] Swann DE, Hass CC, Dalton DC, Wolf SA (2004) Infrared-triggered cameras for detecting wildlife: an evaluation and review. Wildl Soc Bull 32: 357–365.

[cow028C152] Toft G, Baatrup E (2001) Sexual characteristics are altered by 4-*tert*-octylphenol and 17*β*-estradiol in the adult male guppy (*Poecilia reticulata*). Ecotoxicol Environ Saf 48: 76–84. 11161681 10.1006/eesa.2000.1985

[cow028C153] Tolba MK (1992) Saving Our Planet: Challenges and Hopes. Chapman & Hall, London.

[cow028C154] Turner W, Spector S, Gardiner N, Fladeland M, Sterling E, Steininger M (2003) Remote sensing for biodiversity science and conservation. Trends Ecol Evol 18: 306–314.

[cow028C155] Tyler C, Jobling S, Sumpter J (1998) Endocrine disruption in wildlife: a critical review of the evidence. Crit Rev Toxicol 28: 319–361. 9711432 10.1080/10408449891344236

[cow028C156] Valko M, Morris H, Cronin M (2005) Metals, toxicity and oxidative stress. Curr Med Chem 12: 1161–1208. 15892631 10.2174/0929867053764635

[cow028C158] Velando A, Beamonte-Barrientos R, Torres R (2006) Pigment-based skin colour in the blue-footed booby: an honest signal of current condition used by females to adjust reproductive investment. Oecologia 149: 535–542. 16821015 10.1007/s00442-006-0457-5

[cow028C159] von Schantz T, Bensch S, Grahn M, Hasselquist D, Wittzell H (1999) Good genes, oxidative stress and condition-dependent sexual signals. Proc Biol Sci 266: 1–12. 10081154 10.1098/rspb.1999.0597 PMC1689644

[cow028C160] Ward JL, Blum MJ (2012) Exposure to an environmental estrogen breaks down sexual isolation between native and invasive species. Evol Appl 5: 901–912. 23346234 10.1111/j.1752-4571.2012.00283.x PMC3552407

[cow028C161] West PM, Packer C (2002) Sexual selection, temperature, and the lion's mane. Science 297: 1339–1343. 12193785 10.1126/science.1073257

[cow028C162] White AE, Cristol DA (2014) Plumage coloration in belted kingfishers (*Megaceryle alcyon*) at a mercury-contaminated river. Waterbirds 37: 144–152.

[cow028C163] Ying G-G, Williams B, Kookana R (2002) Environmental fate of alkylphenols and alkylphenol ethoxylates – a review. Environ Int 28: 215–226. 12222618 10.1016/s0160-4120(02)00017-x

[cow028C164] Zahavi A (1975) Mate selection—a selection for a handicap. J Theor Biol 53: 205–214. 1195756 10.1016/0022-5193(75)90111-3

[cow028C165] Zala SM, Penn DJ (2004) Abnormal behaviours induced by chemical pollution: a review of the evidence and new challenges. Anim Behav 68: 649–664.

